# Role of UCP2 in the Energy Metabolism of the Cancer Cell Line A549

**DOI:** 10.3390/ijms24098123

**Published:** 2023-05-01

**Authors:** Jessica Segalés, Carlos Sánchez-Martín, Aleida Pujol-Morcillo, Marta Martín-Ruiz, Patricia de los Santos, Daniel Lobato-Alonso, Eduardo Oliver, Eduardo Rial

**Affiliations:** 1 Centro de Investigaciones Biológicas Margarita Salas, CSIC, Ramiro de Maeztu 9, 28040 Madrid, Spain; jsdbmc@ibmb.csic.es (J.S.); carlos.sanchezmartin@uniba.it (C.S.-M.); aleidapujol@mednet.ucla.edu (A.P.-M.); martamartinruiz@atbsarc.org (M.M.-R.); patricia.santos@cib.csic.es (P.d.l.S.); daniel.lobato@cib.csic.es (D.L.-A.); eduardo.oliver@cib.csic.es (E.O.); 2 Centro de Investigación Biomédica en Red de Enfermedades Cardiovasculares (CIBERCV), 28029 Madrid, Spain

**Keywords:** UCP2, cancer, Warburg, uncoupling, reactive oxygen species, proliferation

## Abstract

The uncoupling protein UCP2 is a mitochondrial carrier for which transport activity remains controversial. The physiological contexts in which UCP2 is expressed have led to the assumption that, like UCP1, it uncouples oxidative phosphorylation and thereby reduces the generation of reactive oxygen species. Other reports have involved UCP2 in the Warburg effect, and results showing that UCP2 catalyzes the export of matrix C4 metabolites to facilitate glutamine utilization suggest that the carrier could be involved in the metabolic adaptations required for cell proliferation. We have examined the role of UCP2 in the energy metabolism of the lung adenocarcinoma cell line A549 and show that UCP2 silencing decreased the basal rate of respiration, although this inhibition was not compensated by an increase in glycolysis. Silencing did not lead to either changes in proton leakage, as determined by the rate of respiration in the absence of ATP synthesis, or changes in the rate of formation of reactive oxygen species. The decrease in energy metabolism did not alter the cellular energy charge. The decreased cell proliferation observed in UCP2-silenced cells would explain the reduced cellular ATP demand. We conclude that UCP2 does not operate as an uncoupling protein, whereas our results are consistent with its activity as a C4-metabolite carrier involved in the metabolic adaptations of proliferating cells.

## 1. Introduction

Uncoupling proteins (UCPs) are mitochondrial carriers belonging to the SLC25 family of transport proteins [1]. The name “uncoupling protein” should imply that the biochemical activity of these carriers should reduce the efficiency of oxidative phosphorylation (OXPHOS). The uncoupling activity of UCP1 is well established and provides the molecular basis for heat production in brown adipose tissue [2]. Although UCP2 (SLC25A8) is a carrier that was initially named as an “uncoupling protein” due to its sequence homology to UCP1 [3], its transport activity and physiological roles are still under debate [4,5,6,7,8,9,10,11,12]. UCP2 is expressed in a variety of cell types and its expression levels have often been linked to the defense against oxidative stress. Thus, uncoupling OXPHOS should, in principle, lead to an increase in the rate of respiration that should result in a lower generation of reactive oxygen species (ROS) at the mitochondrial respiratory chain. However, the uncoupling activity of UCP2 and its role in the control of ROS formation remain controversial [4,5,7,8,11,12].

The upregulation of UCP2 in tumor cells has raised considerable attention [13,14,15,16,17,18,19,20]. In addition to its possible role in controlling ROS generation and chemoresistance, it has been proposed that UCP2 could also be involved in the metabolic reprogramming of the cancer cell. UCP2 has been involved in two events related to the Warburg effect: the use of glutamine as an energetic fuel and the export of Krebs cycle C4-metabolites out of mitochondria to be used in the biosynthesis of macromolecules [6,9,10]. It has been proposed that this UCP2-mediated export of C4 metabolites would also reduce the redox pressure on the respiratory chain, thereby reducing ROS formation [9].

Here, we investigate the effect of UCP2 silencing on the energy metabolism of the human lung adenocarcinoma cell line A549. We report that silencing decreases respiration, while state 4 rates (respiration in the absence of ATP synthesis) remain unchanged. The decrease in OXPHOS is not accompanied by a compensatory increase in glycolysis, while the cellular energy charge is maintained. These combined effects reflect a reduced cellular demand for ATP, which could be explained by the observed decrease in cell proliferation and the changes in the expression of proliferation-related genes. We conclude that UCP2 does not have uncoupling activity, whereas our data support its proposed involvement in the metabolic adaptations associated with the Warburg effect.

## 2. Results

### 2.1. OXPHOS and Aerobic Glycolysis

**Figure 1:**
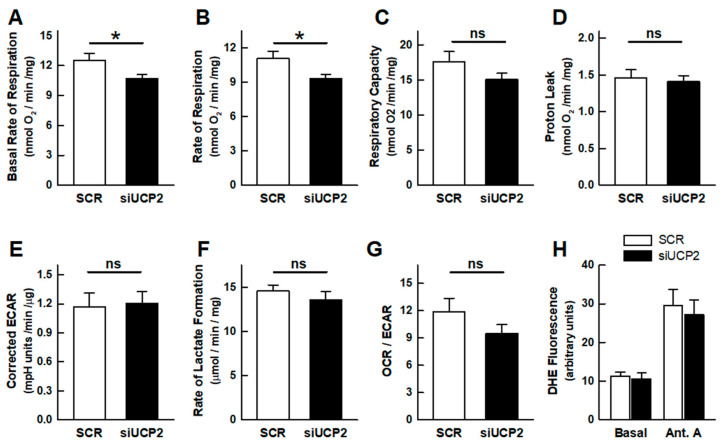
Effect of UCP2-silencing on the energy metabolism of the A549 cell line. (**A**) Basal rate of respiration; (**B**) oligomycin sensitive respiration (ATP turnover); (**C**) uncoupled respiration (maximal respiratory capacity); (**D**) oligomycin-insensitive respiration (proton leakage rate); (**E**) basal rate of glycolysis corrected to take into account the CO_2_ contribution (see methods); (**F**) rate of lactate formation; (**G**) OCR/ECAR ratio (bioenergetic profile); (**H**) DHE fluorescence. Experiments were performed 48 h after transfection. In panels (**A**–**D**,**G**), bars represent the mean ± SEM of 9 independent experiments with 3–5 technical replicates. Bars in panels (**F**,**H**) represent the mean ± SEM of 16 and 5 independent determinations, respectively. Statistical significance: * *p* < 0.05; “ns”: no significant difference.

The silencing of UCP2 expression (Figure S1) alters the cellular energy metabolism, as determined by the analysis of the oxygen consumption rate (OCR) and the extracellular acidification rate (ECAR, a proxy for lactate formation). The parameters calculated using the so-called “mitochondrial stress test” are summarized in Figure 1. Significant differences were observed in the basal respiratory rate and in the ATP-turnover, which were lower in the UCP2-silenced cells (Figure 1A,B). The respiratory capacity (OCR in the presence of the uncoupler FCCP) was 40% higher than the basal rate (Figure 1C). Although an apparent lower respiratory capacity was observed in UCP2-silenced cells, the difference did not reach statistical significance (*p* = 0.152). This result would imply that the mitochondrial mass remains almost unchanged. Interestingly, no differences were observed in the proton leakage rate (OCR in the presence of oligomycin, Figure 1D). A change in the proton leak at a high membrane potential would be expected if silencing affected the proton transport activity of an uncoupling protein [21].

In experiments using the Agilent-Seahorse Extracellular Flux Analyzer, the rate of aerobic glycolysis (lactate formation) can be estimated from ECAR values. As bicarbonate formation from CO_2_ is also a net contributor to extracellular acidification, the CO_2_ contribution to ECAR was estimated as described in the Methods section (Figure S2) to better determine the net rate of lactate formation. The validity of the approach was confirmed by the enzymatic determination of the lactate formation under a variety of experimental conditions and the comparison with ECAR determinations performed in parallel (Figure S2E). The examination of the effect of UCP2 knockdown on the rate of aerobic glycolysis, with the corrections mentioned above, revealed the absence of a significant increase in ECAR (Figure 1E), indicating an unchanged rate of aerobic glycolysis, which was confirmed by the enzymatic determination of the lactate concentration in the culture medium (Figure 1F). The bioenergetic profile of the cells, derived from the ratio between the basal rates of respiration and glycolysis (OCR/ECAR ratio), suggested that the UCP2-silenced cells were slightly more glycolytic, although the difference did not reach statistical significance (*p* = 0.202) (Figure 1G).

### 2.2. Reactive Oxygen Species

It has been proposed that the activity of the UCPs modulates the formation of mitochondrial ROS. If the silencing of UCP2 reduces the respiratory rate, an increase in mitochondrial ROS formation would be expected. Figure 1H shows that silencing did not alter ROS levels as measured by the fluorescent probe DHE. The results with the complex III inhibitor antimycin A, used as a control, showed that under our experimental conditions it is possible to detect an increase in ROS levels upon the inhibition of respiration.

### 2.3. Adenine Nucleotide Levels

**Figure 2:**
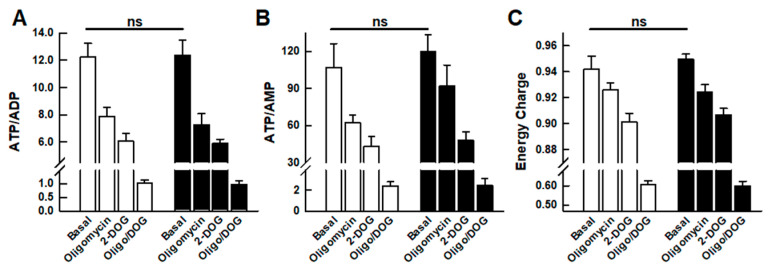
Effect of 48 h UCP2 silencing on the adenine nucleotide levels of the A549 cell line. (**A**) ATP/ADP ratio; (**B**) ATP/AMP ratio; (**C**) energy charge as defined in [22]. Nucleotide extracts were prepared from control A549 cells (open bars) and UCP2-silenced cells (black bars) either untreated (“Basal”) or treated for 30 min with 1 μM oligomycin, 100 mM 2-DOG, or 1 μM oligomycin plus 100 mM 2-DOG (Oligo/DOG). Bars represent the mean ± SEM of 10–14 independent experiments. “ns” denotes the absence of significant differences between “Basal” values.

The combination of a lower respiratory rate without a compensatory increase in aerobic glycolysis reflects a lower energy metabolism in the silenced cells, which could be the result of three possible situations: (i) an increased metabolic efficiency, (ii) a lower cellular energy demand, or (iii) a metabolic failure that compromises the cellular energy levels. Figure 2 shows the results of the HPLC analysis of the cellular content of adenine nucleotide levels and shows that UCP2-silenced cells had an unchanged cellular energy charge and, therefore, no differences in ADP/ATP or AMP/ATP ratios. These results rule out the possibility of metabolic failure. Interestingly, the adenine nucleotide pools in the two cell types responded identically to the inhibition of the metabolic pathways that maintain energy levels.

### 2.4. Cell Growth

**Figure 3:**
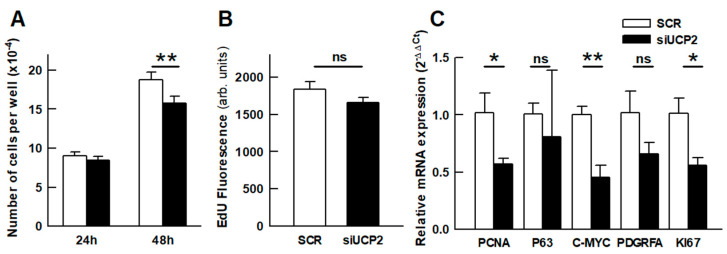
Effect of UCP2 silencing in the proliferation of the A549 cell line. (**A**) Number of cells per well determined 24 and 48 h after transfection. Bars represent the mean ± SEM of 4 independent experiments with 3 technical replicates in each experiment. (**B**) Incorporation of the fluorescent nucleoside analog EdU over a period of 4 h, 48 h after transfection. Bars represent the mean ± SEM of 4 independent experiments with 12–16 technical replicates in each experiment. (**C**) Real time RT-PCR results of cell proliferation-related genes 48 h after transfection. Bars represent the mean ± SEM of 3 independent experiments with 2 technical replicates in each experiment. Statistical significance: * *p* <0.05, ** *p* < 0.01; “ns” no significant difference.

Since UCP2 has been involved in the Warburg effect, which occurs in proliferative cells, there was the possibility that the decreased energy demand was due to a lower proliferation. Figure 3 shows the effect of UCP2 silencing in the proliferation of the A549 cell line. The results revealed a significant decrease in the number of cells 48 h after transfection (*p* = 0.003). The rate of incorporation of the nucleoside analog EdU over a period of 4 h (Figure 3B) followed a trend consistent with the decrease in cell number, although it did not reach statistical significance (*p* = 0.114). The analysis of the expression of five genes related to cell proliferation (PCNA, P63, C-MYC, PDGFRA, and KI67) [23] showed a generalized downregulation that reached statistical significance in three of them, supporting the observed reduction in growth (Figure 3C).

### 2.5. Chromane Derivatives

**Figure 4:**
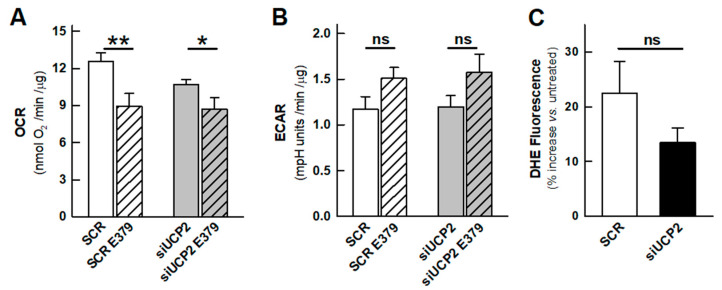
Effect of the chromane derivative CSIC-E379 in control and UCP2-silenced cells. (**A**) Basal rate of respiration and (**B**) basal rate of glycolysis of control cells (SCR) and UCP2-silenced cells treated for 24 h with 20 μM CSIC-E379. Bars represent the mean ± SEM of 6–8 independent experiments with 3–5 technical replicates. (**C**) DHE fluorescence increase in control and UCP2-silenced cells after cell treatment for 24 h with 20 μM CSIC-E379 in 5 independent experiments. Values are expressed as the percentage of fluorescent increase with respect to untreated cells. Experiments were performed 48 h after transfection. Statistical significance: * *p* < 0.05, ** *p* < 0.01; “ns” no significant difference.

We have previously described a set of chromane derivatives that inhibit the proton conductance of yeast mitochondria expressing UCP1 [24]. We also showed that, in HT-29 colon carcinoma cells, the chromane CSIC-E379 inhibited mitochondrial respiration, causing oxidative stress and reducing their viability. We have reviewed the effects of the chromane derivative CSIC-E379 in our new cellular model in which we can test its effect in UCP2-silenced cells. The results showed that the chromane inhibited cellular respiration even in the absence of UCP2 (Figure 4A) and that the inhibition led to (i) an increase in ROS formation (Figure 4C) and (ii) an increase in lactate formation (Figure 4B). These two combined effects indicate a non-specific inhibitory effect of the chromane on the mitochondrial respiratory chain. These toxic effects had not been detected in the screening experiments that led to the identification of these novel inhibitors of UCP1 and UCP2 [24]. For example, the chromane CSIC-E379 had no effect on mitochondria isolated from S. cerevisiae that did not express either UCP1 or UCP2. Since these mitochondria do not possess a proton-translocating rotenone-sensitive NADH dehydrogenase, we suggest that the observed respiratory inhibition may be due to the interaction of the chromane with the mammalian complex I.

## 3. Discussion

**Figure 5:**
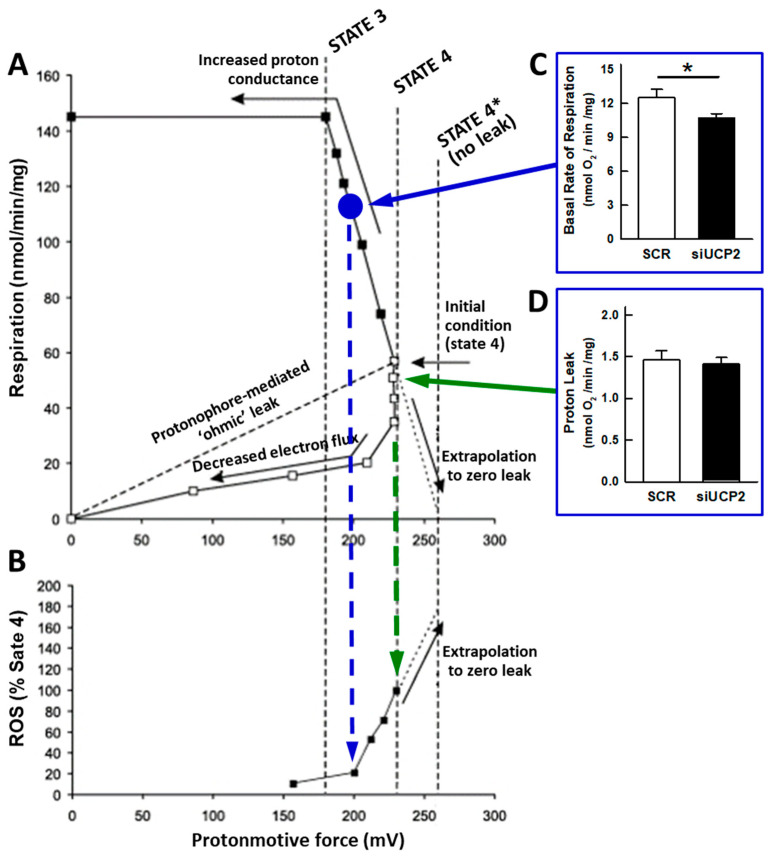
Experimental data relating mitochondrial respiration, protonmotive force, and ROS production (adapted with permission from [37]). (**A**) Relationships between respiration and protonmotive force for brown fat mitochondria that are either exposed to an increasing demand on the proton current (uncoupler titration, closed symbols) or for which substrate supply is progressively restricted (substrate titration, open symbols). State4* indicates the extrapolated value for the protonmotive force in static head conditions, i.e., no proton leak. Original data from [43,44]. (**B**) Data replotted from [33] showing the relationship between H_2_O_2_ generation and protonmotive force. See [37] for further details. Histograms (**C**,**D**) are taken from Figure 1. The observed state 3 rates (**C**) (blue circle would represent 70% of the uncoupled rate) should correspond to low rates of ROS formation (blue dashed arrow). Highest ROS formation would be observed in state 4 (oligomycin present, green dashed arrow) but would not be affected by UCP2 silencing (**D**). * *p* < 0.05.

UCP2 was discovered in 1997 because of its sequence homology to UCP1 [25]. It was found that, while UCP1 was only expressed in brown adipose tissue, UCP2 had a broad tissue distribution [26]. The gene localization in chromosomal regions linked to hyperinsulinaemia and obesity suggested that UCP2 could play a role in the control of energy balance and, in addition, in the response to inflammatory stimuli [25]. The publication in the same year of the leptin induction of UCP2 in white adipose tissue together with enzymes involved in fatty acid oxidation further supported a thermogenic role [27]. The involvement of UCP2 in the control of mitochondrial ROS formation was soon uncovered [28], and, subsequently, reports have continued to emerge pointing to UCP2 as an inducible protein involved in the defense against oxidative stress [4,7,12,14,29,30,31,32]. The mechanistic basis would lie in the steep dependence of the mitochondrial formation of superoxide with membrane potential (Figure 5), such that “mild” respiratory uncoupling or the state 4 to state 3 transition would be sufficient to lower the membrane potential by 10–20 mV and drastically reduce ROS formation [33,34,35,36,37] (Figure 5). These findings suggested that UCP2, like UCP1, was a carrier that would uncouple OXPHOS by increasing proton leakage. However, UCP2-mediated proton permeation has been an elusive biochemical activity that remains controversial [6,8,10,11,20]. Under our experimental conditions, UCP2 silencing did not result in differences in proton leakage, as determined from the rate of respiration in the presence of oligomycin (Figure 1C). Similar results have been reported with isolated mitochondria from UCP2 knockout mice [38,39] and with brain mitochondria from transgenic mice overexpressing UCP2 [40]. The dependence of proton leak on membrane potential [33,38] rules out the possibility of significant UCP2-mediated uncoupling activity if mitochondria are actively phosphorylating ADP.

The presence of UCP2 in tumor cells was another early finding [41] and provides important clues to its biochemical function. The discovery that drug-resistant tumor cells express high levels of UCP2 [14,42], which has been linked to protection against oxidative stress, pointed to UCP2 as a new drug target for cancer treatment [13,15,16,17,24,42].

In addition, an increasing number of reports have implicated UCP2 in the metabolic adaptations of cancer cells, known as the so-called Warburg effect [10,14,18,19,20,45,46,47,48]. However, a clue to this function may lie in the strong regulation of UCP2 translation by glutamine [6,9,49], a key metabolite required for cell proliferation [50,51]. Thus, glutaminolysis provides anaplerotic precursors that drive a glucose-independent Krebs cycle in which intermediates such as citrate or oxaloacetate are exported to be used in the synthesis of macromolecules. In line with these concepts, the discovery that UCP2 is a C4-metabolite carrier that facilitates the export of malate, oxaloacetate, and aspartate from the mitochondrial matrix seems to provide a mechanistic explanation for the role of UCP2 in the metabolic adaptations of cancer cells [9]. In the present work, we have observed that UCP2 silencing decreases the proliferation of the A549 cell line (Figure 3), which is consistent with the proposed involvement of UCP2 in the Warburg effect. Our data suggest that this reduced growth could be the cause of the observed decrease in mitochondrial respiration, which is not compensated by an increase in glycolysis (Figure 1) and does not lead to a decrease in the cellular energy charge (Figure 2). In other words, these results indicate a decrease in cellular ATP demand as a result of reduced cell proliferation.

Our study also provides clues to the role of UCP2 in the control of mitochondrial ROS formation. Under our experimental conditions, the effect of UCP2 silencing is to decrease the basal rate of respiration, i.e., the state 3 rate (Figure 1A). It is important to note the magnitude of the inhibition of basal respiration by oligomycin in control cells (88.3% ± 0.7) and that the FCCP uncoupled rate is only 40% higher than the basal rate, implying that mitochondria are actively synthesizing ATP and should therefore be partially depolarized. The relationship between the rate of respiration, the membrane potential, and the rate of ROS formation shown in Figure 5 (reproduced with permission from [37]) explicitly portrays that, under these conditions, if UCP2 were to catalyze proton leakage it would have no effect on the rate of ROS formation. Accordingly, no differences in DHE fluorescence were observed (Figure 1H). It has already been pointed out that the UCP2-mediated export of matrix C4 metabolites could lower redox pressure on the mitochondrial respiratory chain, leading to reduced ROS production [9], although this would be barely detectable when mitochondria are actively phosphorylating ADP.

As we have previously stated, UCP2 is upregulated in many physiological situations in which there is oxidative stress, and its upregulation has also been linked to the Warburg effect. Proliferating cells, and cancer cells in particular, require metabolic intermediates, reducing power (NADPH) and ATP to synthesize biomass. In addition, cancer cells have to cope with a high intrinsic oxidative stress, and the pentose phosphate pathway is one of the routes that regenerates the NADPH required for ROS detoxification. The increased uptake of glucose and glutamine should serve all these purposes, and cellular metabolism ought to be adjusted accordingly [52,53]. Interestingly, glutamine metabolism has previously been shown to support NADPH production, mainly via the cytosolic malic enzyme [54] and the generation of biosynthetic intermediates. UCP2 would appear to play a key role in these two processes via the export of mitochondrial malate, oxaloacetate, and aspartate [9]. In this context, UCP2 could also be considered an element of the cellular antioxidant defense.

## 4. Materials and Methods

### 4.1. Cell Culture and Materials

Experiments were performed with the cell line A549 (human lung adenocarcinoma) obtained from the American Type Culture Collection (Manassas, VA, USA). Cells were cultured at 37 °C in a humidified 5% CO_2_ atmosphere in standard growth medium: DMEM supplemented with 10% heat-inactivated fetal bovine serum (FBS), 2 mM glutamine, and 100 U/mL of penicillin/streptomycin. All medium components were from Gibco (Life Technologies, Paisley, UK). Culture medium was changed every two days. Cell number and viability were assessed by Trypan Blue (0.2%) staining using a TC10 automated cell counter (Bio-Rad Laboratories, Hercules, CA, USA). Reagents were from Sigma-Aldrich Merck (Darmstadt, Germany) unless otherwise stated.

### 4.2. Silencing

The day before transfection, A549 cells were seeded at a density that led to 50% confluence the following day (e.g., 12k cells/well for XF24 plates, 40k for p24 plates, and 80k cells for p60 plates). A validated siRNA against the human UCP2 obtained from Ambion (s14630, Thermo Fisher Scientific, Lafayette, LA, USA) and a negative scrambled control were used to transfect A549 cells using Lipofectamine 2000 (Invitrogen, Thermo Fisher Scientific, Lafayette, LA, USA) following manufacturer’s protocol. RNAs (1 μM final) and lipofectamine were diluted in Opti-MEM, mixed 1:1, and incubated for 20 min at room temperature. Cells, in transfection medium (DMEM supplemented with 10% FBS and 2 mM glutamine without antibiotics), were transfected adding dropwise the siRNA/lipofectamine mix. Final siRNA concentration was 20 nM. The following day, transfection medium was replaced by standard growth medium, and experiments were carried out 24–48 h later depending on the conditions.

### 4.3. Western Blot Analysis

UCP2 expression levels were determined by Western blot analysis as previously described [29]. A total of 40 μg of cellular extracts were resolved by SDS-PAGE, transferred to nitrocellulose membranes, and probed with an anti-UCP2 antibody (Ref. SC-6525, Santa Cruz Biotechnology, Santa Cruz, CA, USA). Immunoblots were developed with the Super Signal West Dura chemiluminescent substrate (Pierce, ThermoScientific, Rockford, IL, USA).

### 4.4. RTqPCR

Total RNA was extracted from cells with QIAzol reagent (Qiagen, Venlo, The Netherlands). The RNA pellet was dissolved in RNase-free water, and concentration was measured in a NanoDrop spectrophotometer (Thermo Fisher Scientific, Wilmington, DE, USA). RNA (2 μg) was transcribed to cDNA using the High Capacity cDNA Reverse Transcription Kit (Applied Biosystems, Life Technologies Corporation, Carlsbad, CA, USA). qPCR was conducted using PowerUp™ SYBR™ Green Master Mix (Thermo Fisher Scientific, Wilmington, DE, USA) by adding 5.4 μL of SYBR Green and 0.3 μL of each orientation primer to each of the 96-well plates. Running was performed in LightCycler 96 (Roche Diagnostics Corporation, Indianapolis, IN, USA) instrument with the following protocol: step 1 (95 °C for 10 min); step 2 (95 °C for 15 s and 60 °C for 1 min x39 cycles); step 3 (65 to 95 °C with 1 °C increment each for 5 s). HPRT and 18S were used as housekeeping genes [55]. Primers for UCP2, for cell proliferation-related genes (PCNA, P63, C-MYC, PDGFRA and KI67), and for the housekeeping genes are detailed in Supplementary Table S1.

### 4.5. Characterization of the Cellular Bioenergetics

An XF24 Extracellular Flux Analyzer (Agilent Technologies, Santa Clara, CA, USA) was used to determine the OCR and ECAR (a proxy for lactate formation) in the cell line A549. A total of 12k cells were seeded in XF24-well microplates (Agilent Technologies) and transfected 24 h later. Assays were carried out 48 h after transfection. One hour prior to OCR and ECAR measurements, the culture medium was carefully removed, wells washed with 1 mL of assay medium, and, finally, 500 μL of assay medium were added. The assay medium consisted of bicarbonate free DMEM medium pH 7.4 (Agilent Technologies, Santa Clara, CA, USA) supplemented with 2% of FBS, 2 mM glutamine, and 5 mM glucose. Cells were maintained for 1 h at 37 °C in a CO_2_-free incubator before the start of the experiment.

The experimental protocol to determine the bioenergetics parameters was essentially as described in [21], which has often been termed as the “mitochondrial stress test”. Basal OCR and ECAR were determined by performing four measurements, and, subsequently, ATP turnover was assessed from the OCR decrease after the addition of oligomycin (1 μM). The maximal respiratory capacity and the spare respiratory capacity (SRC) were calculated from the OCR increase after two consecutive additions of the protonophore carbonyl cyanide p-(trifluoromethoxy)-phenylhydrazone (FCCP) (0.6 and 0.4 μM). Finally, the respiratory chain inhibitors rotenone (1 μM) and antimycin A (1 μM) were added to determine the non-mitochondrial respiration.

ECAR values were corrected estimating the CO_2_ contribution to the ECAR signal from the correlation between the decreases in the OCR and the ECAR upon the addition of 100 mM 2-deoxyglucose (2-DOG). Figure S2A,B show the steps followed to calculate the CO_2_ contribution: The difference in the ECAR value between the addition of 2-DOG and the inhibitors rotenone/antimycin was assigned as the CO_2_ contribution. The correlation between this ECAR value and the decrease in the OCR under the same conditions was used to estimate the CO_2_ contribution under a variety of experimental conditions (Figure S2C) that were compared with those of the lactate values determined enzymatically (Figure S2D). Figure S2E shows that there exists a good correlation between the two experimental approaches (r^2^ = 0.827, *p* < 0.002).

In all cases, at the end of each experiment with the XF24 analyzer, wells were washed with PBS and protein concentration determined by bicinchoninic acid assay using bovine serum albumin as standard.

### 4.6. Lactate Assay

The rate of lactate formation in the culture media was determined enzymatically. Immediately before the assay, growth medium was replaced with DMEM medium pH 7.4 supplemented with 2 mM glutamine, 5 mM glucose, and FBS 2% that had been previously dialyzed against PBS to remove contaminating lactate. Duplicate samples were collected 60 min later. Lactate concentration was determined with a colorimetric assay that follows NADH formation. Reaction buffer contained 1M glycine, 400 mM hydrazine, and 20 mM EDTA pH 9.8. Reaction was started with the sequential addition of 2 mM NAD^+^ and 60 units of lactate dehydrogenase. Absorbance at 340 nm was measured after 60 min using a Varioskan Flash plate reader (Thermo Fisher Scientific, Wilmington, DE, USA).

### 4.7. Determination of Reactive Oxygen Species

ROS levels were determined following the changes in fluorescence using the probe dihydroethidium (DHE). Sample analysis was carried out with an EPICS XL flow cytometer (Coulter, Hialeah, FL, USA) collecting the fluorescence with a 620 nm band pass filter. Tripsinized cells were resuspended in standard growth medium and incubated for 1 h at 37 °C in the dark with 10 μM DHE.

### 4.8. Adenine Nucleotides Measurement

AMP, ADP, and ATP levels were determined by reverse-phase high performance liquid chromatography (HPLC) essentially as described in [56]. A total of 80k cells/well were seeded in p60 cell culture plates and transfected 24 h later. Adenine nucleotide extraction was performed 48 h after transfection. Thirty minutes before the extraction, growth medium was replaced for DMEM medium supplemented with 2% of FBS, 2 mM glutamine, and 5 mM glucose, and cells were incubated for thirty minutes at 37 °C under the different experimental conditions. Nucleotides were quenched by the addition of 660 mM HClO_4_ and 10 mM theophylline. Plates were quickly frozen using liquid nitrogen and subsequently stored at −80 °C. Extracts were homogenized and centrifuged, and supernatants containing the extracted nucleotides were neutralized with 2.8 M K_3_PO_4_ and stored at −80 °C overnight. Samples were thawed and centrifuged, and supernatants were passed through a 0.45 μm filter. Analysis was performed using a Shimadzu Prominence chromatograph (Canby, OR, USA) using a C18 column (Mediterranea SEA18, Teknokroma, Barcelona, Spain). Peaks were identified according to the retention times of standard adenine nucleotides. Peak assignment was confirmed using samples treated with 2 μM oligomycin plus 50 mM 2-DOG in which the AMP and ADP peaks markedly increased. The cellular energy charge was calculated as indicated in [23].

### 4.9. Cell Proliferation

Cell growth was monitored using a TC10 automated cell counter or by measuring the incorporation of nucleoside analog EdU (5-ethynyl-2’-deoxyuridine). For cell counting assays, 40k cells/well were seeded in p24 cell culture cells and transfected 24 h later. Cell counting was performed 24 and 48 h after transfection. The quantification of the nucleoside incorporation was performed with the Click-iT EdU microplate assay (Invitrogen, ThermoFisher Scientific, Lafayette, LA, USA). A total of 5k cells/well were seeded in 96-well cell culture plates and transfected 24 h later. Forty-eight hours after transfection, growth medium was replaced by fresh medium containing 20 μM EdU and mixed on a rotary shaker for two minutes. Cells were incubated for 4 h at 37 °C in a humidified 5% CO_2_ atmosphere, and, subsequently, label incorporation was determined following manufacturer´s instructions. Amplex Red fluorescence was measured in a VarioskanFlash plate reader with the excitation at 568 nm and the emission at 585 nm.

### 4.10. Statistical Analysis

All values are expressed as mean ± SEM. Differences between groups were determined using either two-tailed unpaired Student’s t-tests or one-way ANOVA using the SigmaPlot software version 11.0. Significant differences between groups are indicated as * *p* < 0.05 or ** *p* < 0.01.

## Data Availability

The data presented in this study are available on request from the corresponding author.

## Supplementary Materials

The following supporting information can be downloaded at: https://www.mdpi.com/article/10.3390/ijms24098123/s1.
