# Supplementary material for: Role of UCP2 in the Energy Metabolism of the Cancer Cell Line A549
Source: Int J Mol Sci. 2023 May 1;24(9):8123. doi: 10.3390/ijms24098123 (PMC10179244; doi:10.3390/ijms24098123)
Supplement: Supplementary file 1 [file ijms-24-08123-s001.zip › ijms-2302643-supplementary.pdf]

Table S1. Real-time PCR primers for the analysis of gene expression.

Gene	Locus	Forward primer	Reverse primer	Size (bp)
**PCNA**	NM_002592	CAAGTAATGTCGATAAAGAGGAGG	GTGTCACCGTTGAAGAGAGTGG	126
**KI67**	NM_002417	GAAAGAGTGGCAACCTGCCTTC	GCACCAAGTTTTACTACATCTGCC	151
**C-MYC**	NM_002467	CCTGGTGCTCCATGAGGAGAC	CAGACTCTGACCTTTTGCCAGG	128
**P63**	NM_003722	CAGGAAGACAGAGTGTGCTGGT	AATTGGACGGCGGTTCATCCCT	122
**PDGFRA**	NM_006206	GACTTTCGCCAAAGTGGAGGAG	AGCCACCGTGAGTTCAGAACGC	121
**18S**	NR_003286	ACCCGTTGAACCCCATTCGTGA	GCCTCACTAAACCATCCAATCGG	159
**HPRT**	NM_000194	TTGGATATAAGCCAGACTTTGTTGG	GGCTTTTGTATTTTGCTTTTCCAGTT	140
**UCP2**	NM_003355	GTCCGGTTACAGATCCAAGGAG	AGCCCATTGTAGAGGCTTCG	131

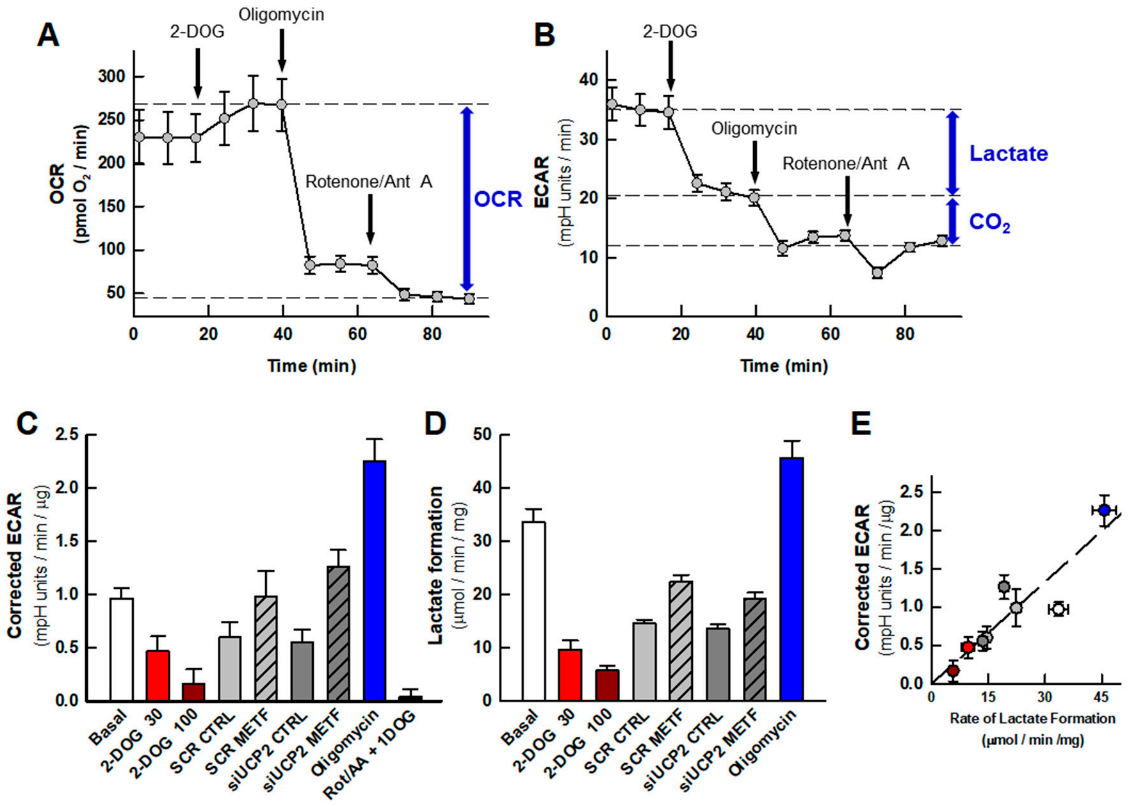

Figure S1. Experimental approach to correct ECAR data and eliminate the CO2 contribution to the acidification and thus better estimate the rate of lactate formation.

(**A**) OCR measurement in A549 cells with the sequential additions of 100 mM 2-DOG, 1 mM oligomycin and 1 µM rotenone plus 1 µM antimycin A. For subsequent calculations, the relevant change in OCR is indicated with the blue double arrow. (**B**) ECAR determination following the same experimental protocol than in (**A**). The ECAR variations that should be due either to lactate or to bicarbonate are indicated. (**C**) Variation in the ECAR signal after the subtraction of the calculated CO_2 contribution with cells under experimental conditions that should lead to changes in lactate formation. (**D**) Rate of lactate formation determined enzymatically as in (**C**).

Experimental conditions for data in panels (**C,D**) are the following: "Basal", untreated A549 cells; "2-DOG 30", A549 cells after treatment with 30 mM 3-DOG; "2-DOG 100", A549 cells after treatment with 100 mM 2-DOG; "SCR CTRL", untreated control A549 transfected cells; "SCR METF" control A549 transfected cells treated for 1 hour with 1 mM metformin; "siUCP2 CTRL", UCP2-silenced A549 untreated; "siUCP2 METF" UCP2-silenced A549 cells treated for 1 hour with 1 mM metformin; "Oligomycin", A549 cells treated with 1 µM oligomycin; "Rot/AA +2DOG", A549 cells treated with 100 µM 2-DOG, 1 µM rotenone and 1 µM antimycin A. (**E**) Correlation between the values presented in histograms (**C,D**) ($r^2 = 0.827$, $p<0.002$).

A

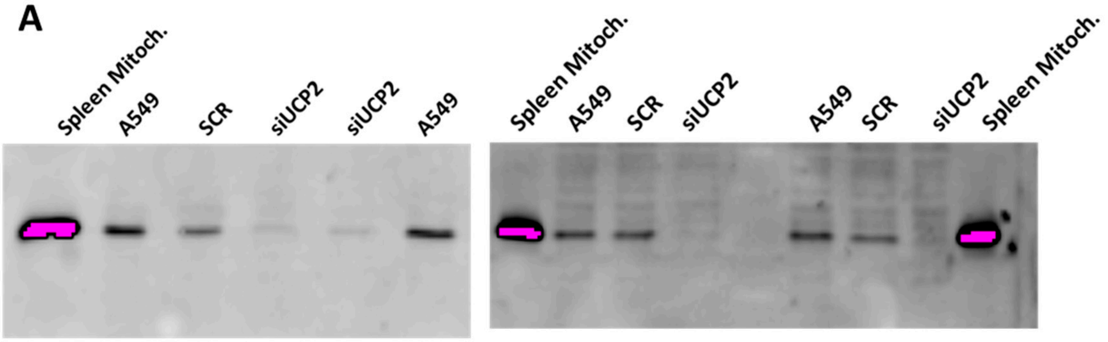

B

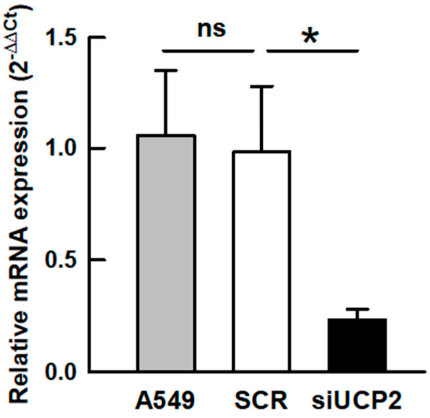

Figure S2. Effect of UCP2 silencing on the protein content and mRNA levels. (**A**) representative Western blots showing the effect of UCP2-silencing on the expression levels of the protein. "Spleen Mit." are mitochondria isolated from mouse spleen used as markers due to the high UCP2 content; A549, cell extracts from non-transfected A549 cells; SCR, cell extracts from control A549 cell transfected with scrambled siRNA. siUCP2, cell extracts from UCP2-silenced cells. Luminescence signal is saturated in some of the lanes to ensure that low UCP2 protein levels can be detected in the rest of the blot. All extracts are from independent cell preparations. (**B**) Real time RT-PCR results of the UCP2 mRNA levels 48 hours after transfection. A549, mRNA from non-transfected A549 cells; SCR, mRNA from control A549 cell transfected with scrambled siRNA. siUCP2, mRNA from UCP2-silenced cells. Bars represent the mean ± SEM of 3 independent experiments with 2 technical replicates in each experiment. Statistical significance: *$p < 0.05$, "ns" not significant difference.
